# A Firmer Understanding of the Effect of Hypergravity on Thyroid Tissue: Cholesterol and Thyrotropin Receptor

**DOI:** 10.1371/journal.pone.0098250

**Published:** 2014-05-27

**Authors:** Elisabetta Albi, Francesco Curcio, Andrea Lazzarini, Alessandro Floridi, Samuela Cataldi, Remo Lazzarini, Elisabetta Loreti, Ivana Ferri, Francesco Saverio Ambesi-Impiombato

**Affiliations:** 1 Laboratory of Nuclear Lipid BioPathology, CRABioN, Perugia, Italy; 2 Department of Clinical and Biological Sciences, University of Udine, Udine, Italy; 3 Institute of Pathologic Anatomy and Histology - University of Perugia, Ospedale Santa Maria Della Misericordia - Piazzale Menghini, Italy; II Università di Napoli, Italy

## Abstract

Maintaining a good health requires the maintenance of a body homeostasis which largely depends on correct functioning of thyroid gland. The cells of the thyroid tissue are strongly sensitive to hypogravity, as already proven in mice after returning to the earth from long-term space missions. Here we studied whether hypergravity may be used to counteract the physiological deconditioning of long-duration spaceflight. We investigated the influence of hypergravity on key lipids and proteins involved in thyroid tissue function. We quantified cholesterol (CHO) and different species of sphingomyelin (SM) and ceramide, analysed thyrotropin (TSH) related molecules such as thyrotropin-receptor (TSHR), cAMP, Caveolin-1 and molecule signalling such as Signal transducer and activator of transcription-3 (STAT3). The hypergravity treatment resulted in the upregulation of the TSHR and Caveolin-1 and downregulation of STAT3 without changes of cAMP. TSHR lost its specific localization and spread throughout the cell membrane; TSH treatment facilitated the shedding of α subunit of TSHR and its releasing into the extracellular space. No specific variations were observed for each species of SM and ceramide. Importantly, the level of CHO was strongly reduced. In conclusion, hypergravity conditions induce change in CHO and TSHR of thyroid gland. The possibility that lipid rafts are strongly perturbed by hypergravity-induced CHO depletion by influencing TSH-TSHR interaction was discussed.

## Introduction

Life on earth has been and is constantly evolving for the variations of different environmental factors, with the exception of the gravitational force that has remained constant over time. The 1gravity(1 g) is responsible for the architecture and function of the cells of all living organisms. So its variation could determine unique and novel effects on cells both *in vitro* and *in vivo* with changes in their biological behavior. Scientific research is mainly devoted to study the effects of microgravity during parabolic flights and longer missions in Space on board the ISS. Nevertheless parabolic flights and spaceflight in general were accompanied by transient hypergravity and vibration [1]. During the initial launch phase, hypergravity forces due to the rocket acceleration were accompanied by launch vibration [1]. In long mission in Space, hypergravity should not be an important factor whereas a certain level of vibration originating from the different machines as well as from the astronauts themselves, for example during workout, was present [2]. Differently, during the parabolic flight each of the 31 parabolas normally flown included 22 s of microgravity and periods of normal and hypergravity [3], [4]. Therefore, the impact of hypergravity on biological systems needs to be considered. In hypergravity conditions plants developed changes in orientation of cortical microtubules and in the metabolism of anti-gravitational cell wall polysaccharides with the modification of body shape and the regulation of cell wall rigidity [5]. Glycoprotein Ib-alpha surface expression and its association with the cytoskeleton were significantly increased in hypergravity-exposed platelets with the increase of their function [6]. In human T cells, ground-based studies to investigate the effect of hypergravity (1.8 g and 9 g) did not reveal any effect on cell cycle control signaling [1]. In myoblast, hypergravity stimulated both proliferation and differentiatiation [7]. It is widely known that musculoskeletal system together to cardiovascular, immune and nervous systems are functionally controlled by the thyroid gland. In follicular thyroid cells mRNA concentrations of growth factors varied significantly under the influence of the mechanical forces generated by hypergravity and vibration [2]. Particularly, in thyroid cells cultured *in vitro* as monolayers IL6 gene activation was very sensitive to physical forces [2]. Changes in the endocrine system and specifically in the thyroid gland represented the main human response to space-flights. We have recently shown structural/functional modifications of thyroid glands isolated from space-flight mice compared to those isolated from control laboratory-kept mice. After long-term exposure to real microgravity environment the thyroid tissues presented a more homogenous structure with prevalence of ordered and large follicles in which thyrocyte cells were thicker with bigger nuclei [8]. Both basal and thyrotropin (TSH)-stimulated cAMP production were higher, while thyrotropin receptor (TSHR) and caveolin-1 were overexpressed [8]. Sphingomyelinase (SMase) and sphingomyelin-synthase (SM-synthase), enzymes involved in cell signaling, were equally overexpressed [9]. It seems that hypogravity and hypergravity would generate opposite results on thyroid gland, but some key results of our previous hypogravity studies were surprisingly similar. In hypergravity the SMase expression did change as in hyporgravity but the nucleus-cytoplasm translocation was similar in the two experimental conditions, suggesting that alteration of gravity conditions might be responsible for molecular remodellings which might influence the cell fate [9]. In addition, both hypogravity and hypergravity induced loss of the parafollicular cells within the thyroid gland, with consequent reduction of calcitonin production, suggesting a potential implication of the mechanical forces in the regulation of bone homeostasis via thyroid gland [10].

Results reported in the literature showed that the effects of hypergravity on the thyroid gland were only partly similar to those produced by hypogravity. In this study we investigated the influence of complex mechanical effects of hypergravity on key proteins and lipids of signal transduction in thyroid gland *in vivo*.

## Materials and Methods

### Reagent

Anti- Signal transducer and activator of transcription-3 (STAT3), anti-TSHR, anti-Caveolin 1, fluorescein isothiocyanate (FITC)-conjugated secondary antibody were obtained from Santa Cruz Biotechnology, Inc. (California, USA); SDS-PAGE molecular weight standards were purchased from Bio-Rad Laboratories (Hercules, CA, USA). Chemiluminescence kits was purchased from Amersham (Rainham, Essex, UK). Cholesterol (CHO), TSH and cAMP EIA kits were purchased from Sigma-Aldrich Corporation (St. Louis, MO, USA) and CABRU SAS (Milan, Italy), respectively. Sphingomyelin (SM) 18∶1 12∶0, SM 18∶1 16∶0, SM 18∶1 18∶1, SM 24∶0, phosphatidylcholine (PC) 16∶0 18∶1, PC 16∶0 20∶4, PC 18∶1 18∶0, ceramide 18∶1 16∶0, ceramide 18∶1 18∶0, ceramide 18∶1 20∶0, ceramide 18∶1 24∶0 were purchased from Avanti (Avanti Polar, Alabaster, USA).

### Experimental design and animal care

All experimental procedures were authorized by the Public Veterinary Health Department of the Italian Ministry of Health. The experiment was also conducted in accordance with the regulations for the care and use of laboratory animals and with the guidelines of the Japanese Physiological Society. Furthermore, this study was also approved by the Committee on Animal Care and Use at Graduate School of Medicine, Osaka University (No. 22-071). Finally, the protocol utilized in the study has been authorized by the Public Veterinary Health Department of the Italian Ministry of Health. All experiments were carried out using male C57BL/10J mice (8 weeks old).

Seven mice were maintained in hypergravity in a 2×g centrifuge (2 gravity samples, 2 g) for 90 days in the laboratory of Dr. Y. Ohira at the Osaka University, Osaka, Japan. Six mice of same strain, treated with the same diet and under the same environmental conditions were maintained at the Vivarium (V samples) of the Advanced Biotechnology Center in Genova, Italy, as control samples. The control mice were similar to those previously used for hypogravity study [9]. Thyroids were sampled bilaterally from each mouse killed by inhalation of carbon dioxide and either processed or frozen immediately, according to the various experimental protocols.

### Thyroid tissue treatment

After excision, right thyroid lobes of all mice under study were used to detect the effect of TSH stimulation on cAMP and TSHR amount. Left lobes of four 2 g mice and three V mice were used for UFLC-MS/MS analysis and left lobes of three 2 g and V mice were used for immunofluorescence analysis.

### TSH stimulation

Right thyroid lobes were divided into 3 fragments: two fragments were treated with 10^−7^ and 10^−8^ M TSH for 1 hour, the untreated fragment was used as control. After stimulation the fragments were fixed with absolute ethanol for 10 min at room temperature and centrifuged for 20 min at 3000×g. The supernatants were used for the evaluation of cAMP levels. At this end the Cayman's cAMP assay Kit, a competitive EIA that permits cAMP measurements within the standard curve range of 0.08–10 pmol/ml, was used as previously reported [11]. The 96-well plates ready to use were supplied in the kit. Each sample was assayed in duplicate at two different dilutions for three times. 50 µl of standard or sample was added to each well. In following steps, performed according to the manufacturing instructions, was first added cAMP acetylcholinesterase (AChE), that is able to bind specific antibodies in inversely proportional way to the free cAMP do dose in the sample, and then was added Ellman's reagent containing the substrate for AChE. The product of the reaction appeared yellow and the intensity of color was measured spectrophotometrically at 412 nm.

The pellets were used to quantify protein amount and for immunoblotting analysis.

### Pellet treatment

The pellets obtained after centrifugation reported in the cAMP assay were homogenized with a dounce homogenizer maintaining temperature at 4°C throughout all procedures. The suspension was used in part for protein dosage [12], in part for immunoblotting analysis by using FRTL-5 cells as potitive control.

### Western immunoblotting

About 30 µg of pellet proteins were submitted to SDS-PAGE electrophoresis in 10% polyacrylamide slab gel for TSHR and STAT3 and 12% slab gel for Caveolin-1 detection. Electrophoresis image analysis was performed on gels stained with Coomassie-blue. Proteins were transferred into nitrocellulose for 90 min as previously described [13]. The membranes were blocked for 30 min with 0.5% no-fat dry milk in PBS (pH 7.5) and incubated overnight at 4°C with the anti-TSHR, STAT3, Caveolin1 specific antibody diluted 1∶1000 with 0.5% no-fat dry milk in PBS. The blots were treated with HRP-conjugated secondary antibodies for 90 min. Visualization was performed with the enhanced chemiluminescence kit. The apparent molecular weight of the proteins was calculated according to the migration of molecular size standards. The area density of the bands was evaluated by densitometry scanning and analysed with Scion Image.

### Lipid extraction

Lipid extraction was performed according to Matyash *et al*. [14]. Pellets of NFL and N were diluted with 1 mL methanol. 3 mL ultra pure water and 3 mL MTBE were added. Each Sample was vortexed for 1 min and centrifuged at 3000 g for 5 min. The supernatant was recovered. The extraction with MTBE was repeated on the pellet and the supernatant was added to the first. The organic phase was dried under nitrogen flow and resuspended in 500 µL of methanol.

### Ultra Fast Liquid chromatography tandem mass spectrometry (UFLC-MS/MS)

Lipid standards SM 18∶1 12∶0, SM 18∶1 16∶0, SM 18∶1 SM 18∶1, PC 16∶0 18∶1, PC 16∶0 20∶4, PC 18∶1 18∶0, ceramide 18∶1 16∶0, ceramide 18∶1 18∶0, ceramide 18∶1 20∶0, ceramide 18∶1 24∶0; and CHO were prepared according to Matyash *et al*., [14]. Standards were dissolved in chloroform/methanol (9∶1 v/v) at 10 µg/mL final concentration. The stock solutions were stored at −20°C. Working calibrators were prepared by diluting stock solutions with methanol to 500∶0, 250∶0, 100∶0, 50∶0 ng/ml final concentrations. 20 µL of standards or lipids extracted from NRL or N samples were injected after purification with specific filter in nylon (0.2 µm).

Analyses were carried out according to Rabagny *et al*. [15] by using Ultra Performance Liquid Chromatography system tandem Mass Spectometer Applaied biosistem (Shimadzu Italy s.r.l., Italy). The lipid species were separated, identified and analysed by following the methods of Rabagny *et al*. [15].

### Immunofluorescence analysis

Tiroid tissues were fixed in 4% neutral phosphate-buffered formaldehyde solution for 24 h. Lobes were dropped with essentially random orientation in paraffin. The paraffin blocks were sectioned into 4-µm-thick sections. All sections were mounted on silan-coated glass slides. Each slide contained a pair of sections at a distance equal to 140 µm. Between 7 and 14 pairs of sections were previously used [9], [10]. In the present study between 15 and 20 pairs of sections were sampled and 17, 18, 19 sections were used for immunofluorescence analysis.

Tissue sections were deparaffinized and rehydrated through a series of xylene and ethanol washes. After 3 washes with phosphate-buffered saline (PBS), sections were incubated with 2 µg/ml anti TSHR primary antibodies diluted in a 0.5% solution of bovine serum albumin in PBS overnight at 4°C. The slides were washed 3 times with PBS and incubated with FITC fluorochrome-conjugated secondary antibodies for 1 hour at room temperature. After 3 washes with PBS, the slides were mounted with glycerol and coverslips. The samples were examined under a fluorescence microscope (OLYMPUS IX 51) equipped with an OLYMPUS DP 50 camera system and analyzed at 20x magnification.

**Statistical analysis. Data were expressed as** means ± SD and their significance was checked by Student 's t-test. P<0.01 versus control samples.

## Results

### 1. Hypergravity up-regulates thyrotropin receptor surface protein but the response to hormonal treatment remains unchanged

**Figure 1:**
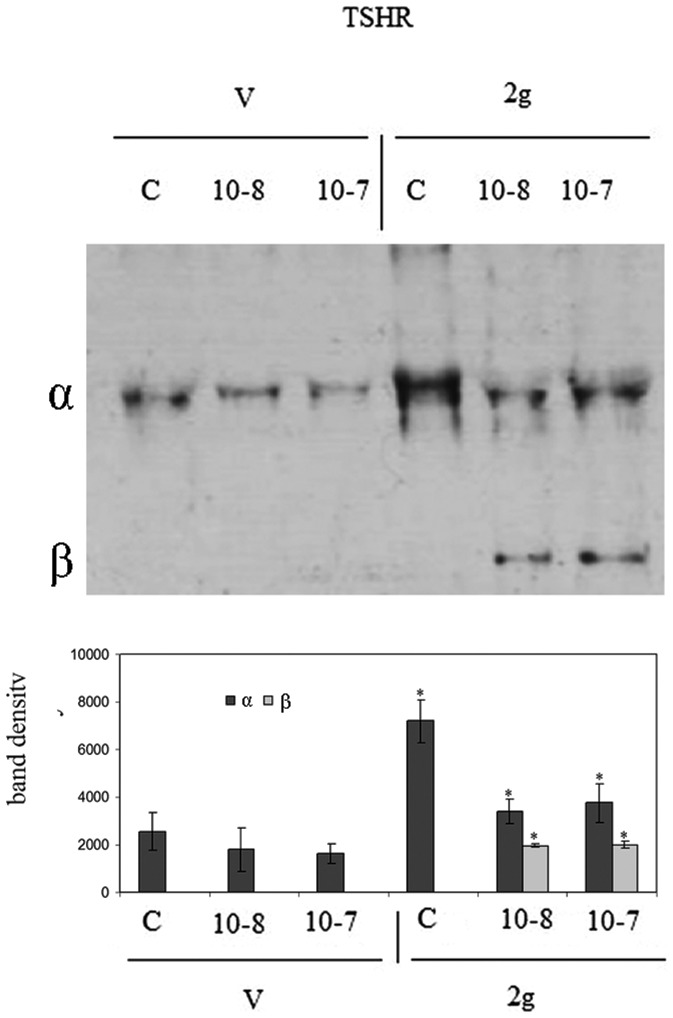
Effect of thyrotropin stimulation on thyrotropin receptor in hypergravity. Thyrotropin receptor in the thyroid tissue of vivarium (V) and 2 gravity (2 g) mice treated or not with 10^−7^ or 10^−8^ thyrotropin (TSH). a) 53 kDa apparent molecular weight of α subunity and 30 kDa of β subunity was indicated in relation to the position of molecular size standard; b) The area density was evaluated by densitometry scanning and analysed with Scion Image programme. The experiment was performed in right lobes of thyroid glands. Data represent the mean ± S.D. of 7 2 g mice and 6 V mice. Significance, *P<0.001 2 g versus V

**Figure 2:**
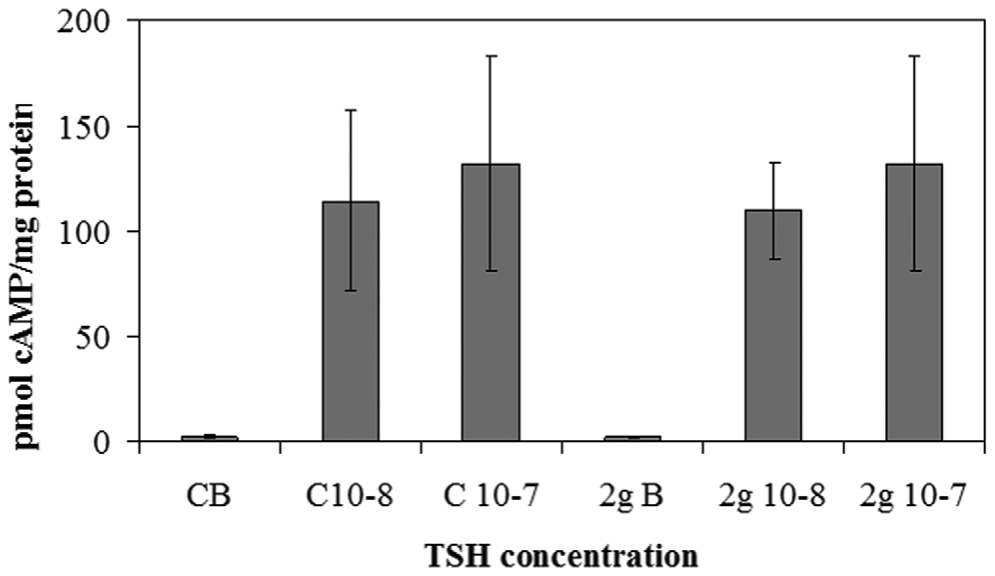
Effect of thyrotropin stimulation on cAMP in hypergravity. cAMP level after 10^−7^ or 10^−8^ thyrotropin (TSH) stimulation in hypergravity. The results were compared with those obtained in vivarium. The experiment was performed in right lobes of thyroid glands The data were expressed as pmol/mg protein and represent the mean ± S.D. of 2gravity (2 g) mice and 6 vivarium (V) mice.

**Figure 3:**
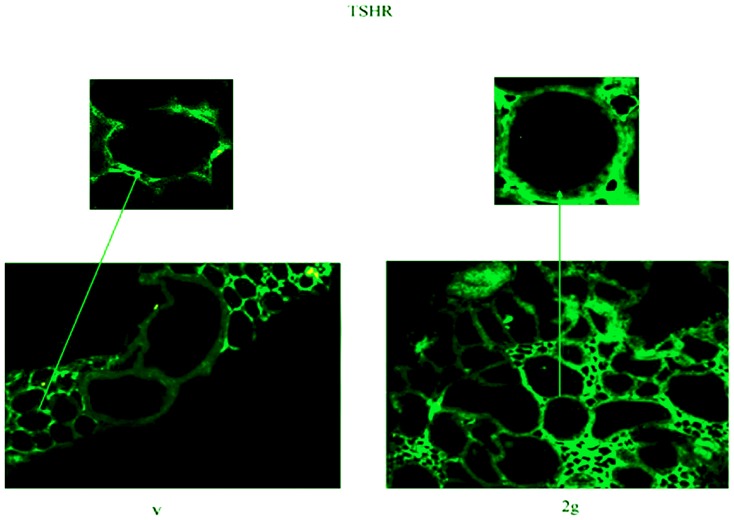
Thyrotropin receptor localization in hypergravity. Fluorescence immunostaining of thyrotropin receptor (TSHR) in thyroid tissues. Analyses were performed using anti-TSHR primary antibody and FITC-conjugated secondary antibody. 20x magnification. The arrows point to the above details of the same areas. The experiment was performed in left lobes of thyroid glands of 3 2gravity (2 g) mice and 3 vivarium (V) mice

**Figure 4:**
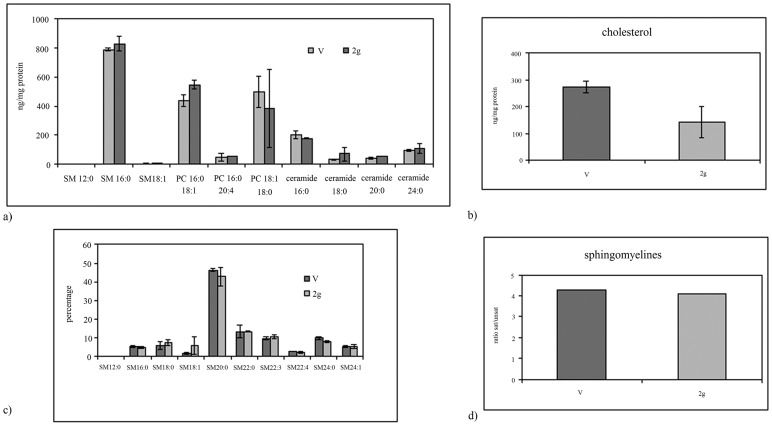
Lipid behavior in hypergravity. Lipid fraction of thyroid tissue in vivarium (V) and hypergravity (2 g) mice. a) amount of specific sphingomyelin (SM), phosphatidylcholine (PC) and ceraminde species; b) cholesterol (CHO) content; c) comparison of areas of different SM species; d) ratio of saturated/unsaturated SM species. The experiment was performed in left lobes of thyroid glands. Data represent the mean ± S.D. of 4 2 g mice and 3 V mice. Significance, *P<0.001 2 g versus V

Experiments of immunoblotting demonstrated that the exposure to hypergravity increased TSHR content (Fig.1a). In the present study the immunoblotting was performed in the pellet obtained with centrifugation after fixation of tissue fragments with absolute ethanol, treatment useful for cAMP evaluation. In the control (C) of both V and 2 g samples we obtained only a single band corresponding to 53 kDa apparent molecular weight (α subunit). The density of the 2 g C band was 2.8 times higher than that of the V C (Fig.1b). 10^−7^ or 10^−8^ TSH treatment did not cause significant changes in the thyroid gland of the V mice (Fig.1a). In contrast, the same treatment in thyroids of mice subjected to 2 g induced about 50% reduction of the α subunit and the appearance of the β subunit, corresponding to 30 kDa apparent molecular weight (Fig.1a, 1b). Thus we have tested TSH-stimulated cAMP production to verify the sensitivity of the thyroid cells to hormone in hypergravity conditions. The results showed a similar basal and TSH-stimulated cAMP production in V and 2 g samples, indicating that even if the TSHR was up-regulated in hypergravity, the loss of its α subunit that links TSH was responsible for the lack of the expected response to hormonal stimulation (Fig.2). It is possible that hypergravity induces a membrane remodelling so that the TSH treatment might facilitate an easier extraction of the β subunit, despite the ethanol treatment. It is therefore possible that perception of hypergravity from the cell membrane may be the first step in the cell response. To verify this hypothesis we performed immunofluorescence analysis of TSHR with specific antibody. The results demonstrated that in V sample the receptor was present on the surface of thyrocytes that surrounded follicles with a precise location, as shown by the sharp brightness of the fluorescent signal (Fig.3). In 2 g samples the fluorescence levels were higher, supporting the data obtained with immunoglotting. The fluorescent signal was also spread over the entire surface of thyrocytes (Fig.3). Since TSHRs were G protein-coupled receptors [16] which were localized in membrane microdomains, including caveolae and lipid rafts enriched in SM and CHO content [17], [18], we next considered if the distribution of TSHR over the entire cell surface in hypergravity conditions could be due to the alteration of lipid component of cell membrane. Thus we performed a UFLC-MS/MS study on lipid fraction of thyroid tissue focusing our attention on SM species and CHO. We have first analyzed the amount of specific species of saturated and mono-unsaturated fatty acids SM, PC and ceramide in order to establish possible specific variation of SM species by using specific standards. Our results showed that not only PC and ceramide species, but also SM species remained unchanged (Fig.4a). Differently, the level of CHO reduced 49% (Fig.4b). Considering the relationship SM-CHO known in the literature, we extended our observation to the SM species containing longer fatty acid chains and with greater degrees of unsaturation. The identification of the molecules was carried out according to the RT and the molecular weight. The comparison between V and 2 g samples was carried out by calculating the percentage of the areas. No significant changes were found for each SM species (Fig.4c) and for saturated/unsaturated ratio of total SM (Fig.4 d).

### 2. Gravity controls structural/functional proteins

**Figure 5:**
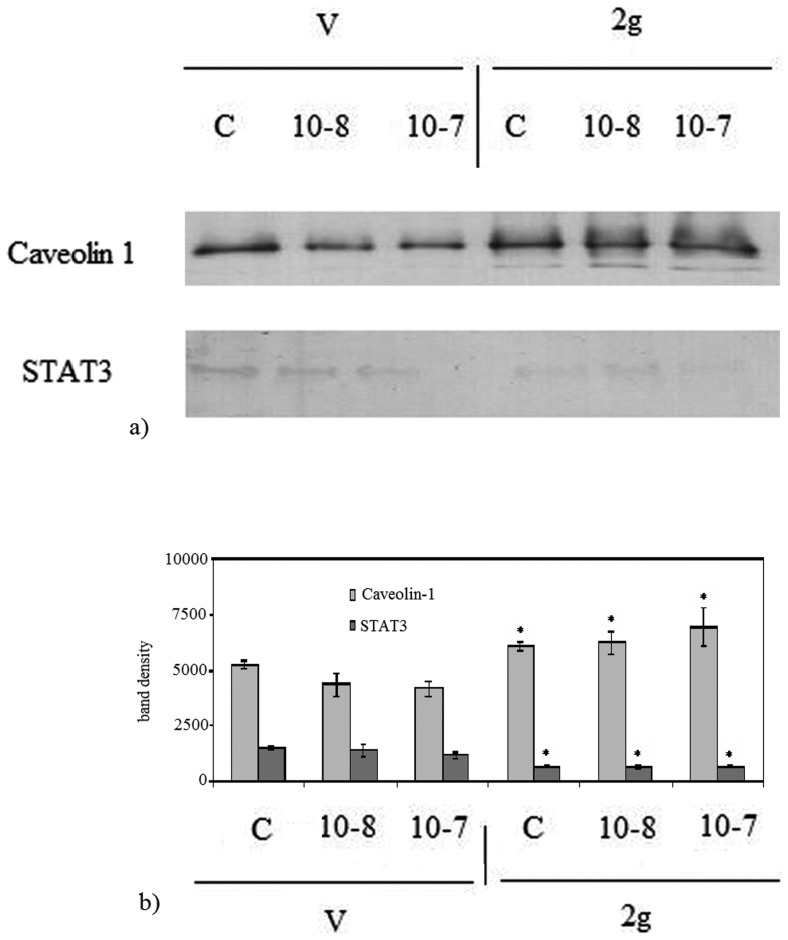
Effect of hypergravity on Caveolin 1 and STAT3 proteins. Caveolin 1 and STAT3 in the thyroid tissue of vivarium (V) and 2 gravity (2 g) mice treated or not with 10^−7^ or 10^−8^ thyrotropin (TSH). a) bands of 22 kDa apparent molecular weight for Caveolin 1 and 90 kDa for STAT3 was indicated in relation to the position of molecular size standard; b) The area density was evaluated by densitometry scanning and analysed with Scion Image programme. The experiment was performed in right lobes of thyroid gland Data represent the mean ± S.D. of 7 2 g mice and 6 V mice. Significance, *P<0.001 2 g versus V.

To explore the mechanisms underlying the effect of gravity on thyroid protein fraction, we examined proteins relevant for the thyroid function; Caveolin-1 which is a critical protein to caveolae and STAT3 which is a suppressor of thyroid tumor growth [19]. Our results showed the increase of Caveolin-1 protein in both TSH-untreated and TSH treated thyroid tissues of 2 g mice in comparison with V mice (Fig. 5a). In fact, the density value of Caveolin-1 band, corresponding to 22 kDa apparent molecular weight, was 14% higher in 2 g C than V C and it did not change significantly after TSH treatment (Fig.5b). Immunopositivity of STAT3 was reduced in 2 g mice in comparison with V mice (Fig.5a). The area density of STAT3 protein, corresponding to 90 kDa apparent molecular weight, was 53% lower in 2 g C than V C and it unchanged significantly after TSH treatment (Fig.5b).

## Discussion

Based on our previous results of hypergravity-challenged thyroid glands [9], [10] we had hypothesized that under hypergravity conditions thyrocytes might remodel their cell membrane by increasing TSHR surface protein and consequently change their sensitivity to TSH treatment. Our results showed that this relationship is not so straightforward. TSHR is a G protein-coupled receptor [16]. It is a heterodimer [20] that includes an α extracellular subunit (53 kDa), that interacts with TSH, and a broad β transmembrane and intracellular subunit (30–42 kDa), held together by disulphide bridges [21]. Costa *et al*. [22] demonstrated that TSHRs were not distributed randomly in cell membranes but were rather localized in the basolateral membrane rich in lipid rafts, microdomains with high level of CHO and sphingolipids [23]. Differently, Latif *et al*. [24] demonstrated that monomeric and multimeric TSHRs were present in cell surface in both raft and no-raft fractions as caveolae. However the raft domains provided a platform for the assembly of signaling complexes of receptors [25]. Our results showed that hypergravity up-regulated 65% TSHRs and only 14% Caveolin-1. In addition, treatment with TSH in 2 g samples strongly changed the TSHR but had no effect on Caveolin-1. We thus suggest that gravity forces may remodel cell membrane structure by acting specifically on lipid rafts. Loosfelt *et al*. [20] indicated that TSH treatment facilitated the shedding of α subunit of TSHR and its releasing into the extracellular space. We demonstrated that TSH treatment did not induce a significant reduction of the TSHR α subunit in V mice, whereas it was reduced 2.1 times in 2 g mice. In C V mice, after TSHR treatment we would have expected two bands of α and β subunits and an evident reduction of the α subunit. It is useful to consider in this regard that we have carried out the evaluation of the TSHR in the pellet obtained after treatment of the tissue with ethanol necessary for the analysis of the production of cAMP. So it is possible that the ethanol had a fixative effect on the membranes by impairing the shedding of TSHR α subunit after TSH treatment and the extraction of the intramembrane β subunit. The behavior of TSH subunits in hypergravity samples was very different. Based on these findings, our opinion was that lipid rafts were strongly perturbed by hypergravity-induced CHO depletion, so that the TSHR lost its specific localization and spread throughout the cell membrane, as demonstrated by immunofluorescence. It was likely that the spatial relationship of the two subunits could be amended during the movement in such a way that the treatment with TSH could induce shedding of α subunit and extraction of β subunit, despite treatment with ethanol. The results presented in this paper have been really intriguing to us because only two years ago we obtained similar results on thyrocytes cultured under conditions of microgravity [11]. In that experiment FRTL-5 thyroid cell line was exposed to microgravity during the Texus-44 mission. In such circumstance, our *in vitro* system had allowed us to demonstrate the presence of the TSHR and the increase of CHO in the culture medium. In hypogravity the cAMP production after TSH stimulation was inhibited with respect to control samples [11]. Here instead we observed similar results in hypergravity and in control samples. Such difference could be due to the upregulation of the TSHR in hypergravity. Microgravity also induced the release of total SM measured by thin layer chromatography. Here with a more sophisticated technique we did not notice any significant variation of individual SM species. On the other hand we had already shown that the enzymes responsible for SM metabolism behaved similarly under hypo- and hypergravity conditions [9]. In hypergravity, no variation of SMase and SM-synthase1 expression was found as in hypogravity but the SMase translocated from the nucleus to the cytoplasm and had similar values of enzyme activity to those in hypogravity [9]. Comparing the results from hypo-and hypergravity, it was evident that the plasma membrane was significantly altered either increasing or decreasing the mechanical load of the gravitational force. CHO removal perturbed lipid rafts that act as platform for TSHR. Therefore membrane CHO and not SM was critical for TSH-TSHR interaction. Depletion of membrane CHO by methyl-beta-cyclodextrin resulted in a disruption of lipid rafts in plasma membrane [26] whereas extensive sphingolipid depletion did not affect lipid raft integrity [27]. Rapid changes of the cytoskeleton as reaction to gravitational changes were reported in diverse cell types [28], [29] and they could be responsible for changes in surface receptors [1]. Here we reported the first observation of altered receptor by perturbation of membrane CHO.
